# Assessing Serum Lipid Levels in Patients Diagnosed With Primary Open-Angle Glaucoma

**DOI:** 10.7759/cureus.74582

**Published:** 2024-11-27

**Authors:** Manish K Karn, Madhulika Sinha, Deepak Mishra, Abhishek Anand

**Affiliations:** 1 Ophthalmology, Indira Gandhi Institute of Medical Sciences, Patna, IND; 2 Ophthalmology, Institute of Medical Sciences, Banaras Hindu University, Varanasi, IND

**Keywords:** dyslipidemia, intraocular pressure, lipid peroxidation, primary open-angle glaucoma, serum lipids

## Abstract

Purpose of the study: The main purpose of this study is to assess serum lipid levels in patients diagnosed with primary open-angle glaucoma (POAG). The secondary objective is to explore the association between these lipid levels, namely serum cholesterol, serum triglycerides, serum low-density lipoprotein (LDL), and serum high-density lipoprotein (HDL), and the development of POAG.

Background: Glaucoma is a major contributor to global blindness, with elevated intraocular pressure as a key risk factor. However, in some cases, the disease advances even when pressure levels are well controlled, suggesting that additional independent risk factors may play a role in its development. Previous epidemiological studies have shown a potential link between serum lipids and glaucoma, but the findings are contradictory, leading to this investigation into the relationship between serum lipid levels and POAG.

Materials and methods: The study involved 60 patients with POAG and a control group of 60 individuals without POAG. All individuals underwent a detailed ophthalmic examination, and fasting serum lipid levels were measured, including total serum cholesterol, serum triglycerides, LDL, and HDL. The lipid levels of the glaucoma group were then compared to those of the control group.

Results: The findings revealed that total cholesterol, triglycerides, and LDL levels were significantly higher in POAG patients compared to the control group (p < 0.05). Although HDL levels were lower in POAG patients, the difference was not statistically significant.

Conclusion: Dyslipidemia appears to be an independent risk factor for POAG, with higher serum lipid levels strongly linked to the presence of the condition.

## Introduction

Primary open-angle glaucoma (POAG) is a progressive, irreversible optic neuropathy that is chronic in nature and the second most common cause of preventable blindness in India. In India, around 112 million people aged 40 and older are affected by glaucoma, with an estimated 6.48 million cases of POAG [1]. Often, the disease goes unnoticed until its later stages, as symptoms typically emerge only when significant damage has occurred. Given the impact of POAG, understanding and identifying the risk factors that contribute to its onset is essential, as multiple factors are believed to influence its development. The primary risk factor for glaucoma is high intraocular pressure (IOP), which can damage the optic nerve either by causing direct mechanical injury to the retinal nerve fiber layer or by restricting blood flow to the optic nerve head, leading to ischemic damage [2]. Nonetheless, it is widely acknowledged that additional risk factors must also play a role, contributing to the development and progression of glaucoma either by influencing IOP or through alternative mechanisms. POAG has been linked to type 2 diabetes mellitus (DM) and hypertension. Both conditions are often associated with elevated serum lipid levels, as dyslipidemia and insulin resistance are interconnected, and elevated serum lipid levels can contribute to atherosclerotic changes. This suggests a potential indirect relationship between glaucoma and serum lipid levels [3]. The association of triglycerides and glaucoma has been a matter of debate in recent times.

Lipid peroxidation, which leads to oxidative stress, may directly damage the trabecular meshwork and endothelium of blood vessels supplying the optic nerve head. Alternatively, atherosclerotic changes due to elevated serum cholesterol could impair ocular blood flow [4]. Previous research has shown higher levels of lipid peroxides in the aqueous humor, trabecular meshwork, and Schlemm's canal in POAG patients compared to controls, suggesting that lipid peroxidation, through increased oxidative stress, plays a role in damaging these structures. Additionally, elevated lipid levels might raise episcleral venous pressure and blood viscosity, reducing outflow efficiency [5].

Currently, there is a lack of sufficient studies investigating the relationship between dyslipidemia and POAG. Hence, we conducted this study to better understand the potential association between serum lipid levels and the development of POAG.

## Materials and methods

This case-control study was carried out from November 2019 to August 2021 at the Regional Institute of Ophthalmology, Banaras Hindu University, Varanasi, India. A total of 120 participants were included. About 60 patients were diagnosed with POAG based on clinical assessments and standard ophthalmologic examinations, and 60 control participants were patient attendants without POAG or other eye disorders. All eligible cases who met the inclusion and exclusion criteria during the 20-month period were enrolled. The study received approval from the Ethics Committee of the Institute of Medical Sciences, Banaras Hindu University, and was conducted in compliance with the Helsinki Declaration. All participants provided written informed consent.

The study included participants aged 18 and older who had POAG without any additional ocular diseases. The inclusion criteria for glaucoma cases were an untreated IOP of 21 mm Hg or higher measured with a Goldmann Applanation Tonometer, open anterior chamber angles observed on gonioscopy, glaucomatous changes in the optic disc (such as an increased cup-to-disc ratio, neuroretinal rim thinning, or notching) detected on fundoscopy, and visual field defects typical of glaucoma identified using standard automated perimetry with a Humphrey Visual Field Analyzer. For controls, the criteria included an IOP below 21 mm Hg, no glaucomatous changes in the optic disc, and no visual field loss characteristic of glaucoma. Those with a history of angle-closure glaucoma, non-dilating pupils, ocular trauma, other ocular conditions (except refractive errors), or systemic diseases affecting the eyes, such as diabetes and hypertension, intraocular surgery, and cases involving subluxated, traumatic, or complicated cataracts were excluded from this study. Additionally, individuals using lipid-lowering medications like statins were not included.

Demographic information, including age, gender, and address, was recorded. Ophthalmic assessments included visual acuity, anterior segment examination using slit-lamp biomicroscopy, Van Herick’s grading, pupillary reaction, and IOP measurement with an applanation tonometer. The angle structures were evaluated through gonioscopy, while fundus examination and visual field assessment were conducted. The diagnosis of POAG was based on increased IOP, changes in the optic nerve head observed with slit-lamp biomicroscopy using a 78D lens, and visual field abnormalities.

Laboratory assessment

A 5 ml venous blood sample was drawn from the right antecubital vein and collected in an ethylenediaminetetraacetic acid (EDTA) vial following 10-12 hours of overnight fasting to measure serum lipids. The samples were then sent to the Center of Clinical Investigations (CCI) Lab at Sir Sundarlal Hospital, Banaras Hindu University, where they were analyzed using the enzymatic colorimetric method on an autoanalyzer, specifically the Mindray model BS-800 (Mindray Medical India Pvt. Ltd., India). Reference values for serum lipids were based on the National Cholesterol Education Program: Adult Treatment Panel III (NCEP: ATP III) guidelines. According to these standards, hypertriglyceridemia is defined as triglycerides ≥150 mg/dl, hypercholesterolemia as a total cholesterol level of ≥200 mg/dl, low-density lipoprotein (LDL) as high if ≥130 mg/dl, VLDL as high if ≥30 mg/dl, and high-density lipoprotein (HDL) cholesterol as low if ≤40 mg/dl. The mean, standard deviation, and standard error of the mean were evaluated.

Statistical analysis

The data analysis was carried out using SPSS Statistics for Windows, Version 16 (Released 2007; SPSS Inc., Chicago, United States). The results are expressed as mean±standard deviation. An unpaired Student's t-test was used to compare the characteristics of subjects between the groups. All reported p-values less than 0.05 were considered statistically significant, with p-values less than 0.01 or 0.0001 highly significant.

## Results

In our study, a total of 120 participants were included, with 60 in the glaucoma group and 60 in the control group. Participants' ages ranged from 40 to 82 years, with an average age of 58 years in the glaucoma group and 60 years in the control group. The majority of cases and controls fell within the 55-65 age range.

The male-to-female ratio was 2.1:1 in the POAG group and 1.6:1 in the non-POAG group. The mean age was 58.22±6.82 years in the glaucoma group and 60.05±8.41 years in the control group (p=0.543). Of the glaucoma cases, 39 (65%) out of 60 were from urban areas, and 21 (35%) out of 60 were from rural areas. In the control group, 44 out of 60 (73%) were from urban backgrounds, while 16 out of 60 (27%) were from rural backgrounds. There were no statistically significant differences in the age, sex, and locality distributions between the two groups, as confirmed by the t-test results, which showed no notable variation. Demographic characteristics of the study population are presented in Table 1.

**Table 1:** Demographic characteristics of study population *T-test statistics; p-value<0.05 was considered as statistically significant

Characteristics	Cases (n=60)	Controls (n=60)	p-value
Mean age (in years)	58.22±6.82	60.05±8.41	0.543*
Gender			
Male	41 (68.3%)	37 (61.7%)	0.444*
Female	19 (31.7%)	23 (38.3%)	0.444*
Locality			
Urban	39 (65%)	44 (73%)	0.323*
Rural	21 (35%)	16 (27%)	0.323*

In the glaucoma group, the mean cholesterol level was 204.22±40.76 mg/dl, mean triglycerides were 162.25±32.59 mg/dl, mean LDL was 123.71±41.25 mg/dl, mean VLDL was 33.58±9.83 mg/dl, and mean HDL was 39.99±8.74 mg/dl. In the non-glaucoma group, the mean cholesterol level was 162.81±35.15 mg/dl, mean triglycerides were 111.43±14.77 mg/dl, mean LDL was 105.81±16.03 mg/dl, mean VLDL was 25.05±5.90 mg/dl, and mean HDL was 41.75±6.82 mg/dl (Table 2).

**Table 2:** Serum lipid values in cases and controls represented as mean±SD statistical parameter LDL: low-density lipoprotein; HDL: high-density lipoprotein; VLDL: very low-density lipoprotein

Parameters applied	Cases (n=60)	Control (n=60)
Mean cholesterol	204.22±40.76	162.81±35.15
Mean triglycerides	162.25±32.59	111.43±14.77
Mean LDL	123.71±41.25	105.81±16.03
Mean VLDL	33.58±9.83	25.05±5.90
Mean HDL	39.99±8.74	41.75±6.82

Elevated cholesterol levels (>200 mg/dl) were seen in 34 (56.7%) cases, while 7 (11.7%) participants in the control group had high cholesterol. High triglyceride levels (>150 mg/dl) were found in 45 (75%) cases, compared to 11 (18.3%) controls. Raised LDL levels (>130 mg/dl) were present in 33 (55%) cases and 5 (8.3%) in the control group. High VLDL levels (>30 mg/dl) were seen in 36 (60%) cases and 7 (11.7%) in controls. Low HDL levels (<40 mg/dl) were noted in 25 (41.7%) cases and 16 (26.7%) in the control group (Table 3).

**Table 3:** Dyslipidemia in cases and controls *T-test statistics, p-value<0.05 was considered as statistically significant and p<0.01 or p<0.0001 as highly significant; **odds ratio; ***confidence interval

Lipid parameters	Cases (n=60)	Controls (n=60)	p-value^*^	OR^**^	95% CI^***^
High cholesterol	34 (56.7%)	7 (11.7%)	0.0001	9.901	3.870-25.325
High triglycerides	45 (75%)	11 (18.3%)	0.0001	13.363	5.559-32.121
High LDL	33 (55%)	5 (8.3%)	0.0001	13.44	4.714-38.316
High VLDL	36 (60%)	7 (11.7%)	0.0001	11.357	4.425-29.143
Low HDL	25 (41.7%)	16 (26.7%)	0.0852	1.96	0.910-4.236

There was no statistically significant difference in mean age or sex between the POAG and control groups. Mean levels of cholesterol, triglycerides, VLDL, and LDL were significantly higher in the case group compared to the control group, with a p-value of <0.001 at a 95% confidence interval (CI). While HDL levels were lower in the case group than in the control group, this difference was not statistically significant (p=0.083). Logistic regression analysis showed associations between serum lipid levels and POAG after adjusting for age and other demographic factors. HDL had an odds ratio (OR) of 1.96 (95% CI=0.910-4.236, p=0.0852), while LDL had an OR of 13.44 (95% CI = 4.714-38.316, p=0.0001), VLDL an OR of 11.357 (95% CI=4.425-29.143, p=0.0001), triglycerides an OR of 13.363 (95% CI= 5.559-32.121, p=0.0001), and cholesterol an OR of 9.901 (95% CI=3.870-25.325, p=0.0001).

## Discussion

POAG is a prevalent condition that imposes a significant burden on society, making it essential to identify effective prevention strategies. This study examined the relationship between serum lipid levels and POAG. Findings indicated a significant association between POAG and elevated cholesterol, LDL, and triglycerides, as well as low HDL. HDL levels were lower in the case group than in the control group, but this difference was not statistically significant.

In their study, Kovacevic et al. concluded that raised serum lipid levels, especially the atherogenic LDL fraction, could have an effect on the development of glaucoma [6]. Serum lipid levels for triglycerides, HDL, and LDL lipoproteins were similar in both groups, but cholesterol levels were significantly higher in the POAG group.

Egorov et al. found that lipid biochemical analyses in glaucoma cases may indicate atherogenic hyperlipidemia associated with lower antioxidative activity [7]. Long-term use of statins, exceeding 23 months, may significantly reduce the risk of developing glaucoma. Thus, while statins used for hyperlipidemia treatment may not alter IOP in glaucoma patients, they could help reduce the risk of glaucoma [8]. In a case-control study by Davari et al., a positive association was identified between POAG and dyslipidemia, showing an OR of 7.14 (95% CI: 2.3-22.2) for hypercholesterolemia and an OR of 16.9 (95% CI: 2.1-14.8) for hypertriglyceridemia [9]. The study suggested that hyperlipidemia could contribute to the risk of developing POAG. In a 2009 study conducted by Pavljasević and Asćerić [10] in Bosnia and Herzegovina, researchers investigated the serum lipid levels of 50 individuals with POAG and 50 healthy individuals. In the glaucoma group, the mean cholesterol level was 6.14 mol/dm, slightly higher than the control group's mean of 5.96 mol/dm. Similarly, the glaucoma group had a mean triglyceride level of 2.38 mol/dm, while the control group had a lower average of 2.04 mol/dm. High-density cholesterol averaged 1.45 mol/dm in the glaucoma group and 1.40 mol/dm in the control group. Low-density cholesterol was 3.98 mol/dm in the glaucoma group and 4.08 mol/dm in the control group. These findings indicate that blood cholesterol levels were higher in glaucoma patients, suggesting that hypercholesterolemia may be a predictive factor for POAG diagnosis.

In the Beijing Eye Study [11], about 3,251 participants aged 45 and older underwent detailed eye exams, and their blood serum lipid levels were assessed. The study accounted for variables like age, gender, location, income, BMI, smoking status, diastolic blood pressure, and blood glucose levels before exploring how dyslipidemia influenced the onset of eye conditions. The results showed that individuals with dyslipidemia exhibited significantly elevated IOP. The results indicated that patients with dyslipidemia had significantly increased IOP. In a study conducted by Chisholm and Stead [12] involving 183 individuals (92 women and 91 men) with POAG, the researchers aimed to assess serum lipid levels and found that only triglyceride levels were significantly elevated in adult females. In a 1996 study by Stewart et al., a comparison of total cholesterol and HDL levels was made between 25 glaucoma patients and 25 healthy individuals, revealing no association between HDL or total cholesterol levels and IOP or POAG [13].

The association between serum lipids and POAG might be influenced by the disorder's correlation with other cardiovascular risk factors, like diabetes and hypertension. A cohort study at the University of Michigan investigated individuals over 40 years old who had visited an ophthalmologist at least once between 2001 and 2007. This study aimed to investigate the components of metabolic syndrome in relation to glaucoma [14]. Results indicated that both diabetes and hypertension, whether occurring alone or together, play a significant role in the onset of glaucoma. Conversely, dyslipidemia by itself appeared to lower the risk of developing glaucoma by approximately 5%. However, when dyslipidemia was present alongside diabetes or hypertension, the risk for glaucoma increased. This suggests a synergistic effect between dyslipidemia and these conditions in the development of glaucoma. Our findings indicate that dyslipidemia remains an independent risk factor for the development of POAG after controlling for these confounding factors. These studies enhance our understanding of the mechanisms through which dyslipidemia contributes to the onset of POAG. Research has shown elevated levels of lipid peroxides in the aqueous humor, trabecular meshwork, and Schlemm's canal in POAG patients compared to non-glaucoma individuals, suggesting that lipid peroxidation may increase oxidative stress, leading to damage in the trabecular meshwork and Schlemm's canal [15,16].

Limitations of this study

The study was conducted with a relatively small sample size (60 POAG patients and 60 controls) in a single regional hospital, the study’s findings may be influenced by local population characteristics. A larger sample size with a broader demographic representation would strengthen the validity of the results and provide a clearer understanding of the association between serum lipids and POAG across different populations. Being a cross-sectional study, this research establishes association but cannot confirm causation. The study design limits our ability to determine whether dyslipidemia is a contributing factor to POAG development or a consequence of the disease. Longitudinal studies are needed to understand the directionality of this relationship. The study excluded patients on lipid-lowering medications, which helps to reduce bias related to medication effects on lipid profiles. However, this exclusion may also limit the applicability of the results to individuals who are commonly prescribed lipid-lowering therapies and could have benefited from understanding the potential impact of these medications on POAG risk. Addressing these limitations in future studies could provide a more comprehensive understanding of the role of serum lipids in POAG and improve the clinical utility of these findings.

## Conclusions

This study sheds light on the significant association between dyslipidemia and POAG, suggesting that serum lipid profiles may be an essential factor in assessing POAG risk. Our findings show that elevated levels of triglycerides, cholesterol, VLDL, and LDL are prevalent among POAG patients compared to non-glaucomatous individuals. By integrating serum lipid evaluations into glaucoma management, it may be possible to identify at-risk individuals earlier, allowing for more effective preventive measures and therapeutic interventions. In summary, dyslipidemia has been identified as an independent risk factor for POAG. Further, longitudinal studies are essential to explore the exact mechanisms linking lipid dysregulation and optic nerve damage in POAG and to assess whether lipid-lowering therapies could serve as an adjunct to traditional glaucoma treatments.
